# Designing In-House SARS-CoV-2 RT-qPCR Assay for Variant of Concerns

**DOI:** 10.1055/s-0042-1756660

**Published:** 2022-09-20

**Authors:** Mahmut Cerkez Ergoren, Gulten Tuncel, Cenk Serhan Ozverel, Tamer Sanlidag

**Affiliations:** 1 Department of Medical Genetics, Faculty of Medicine, Near East University, Nicosia, Cyprus; 2 DESAM Research Institute, Near East University, Nicosia, Cyprus; 3 Department of Basic Medical Sciences, Faculty of Dentistry, Near East University, Nicosia, Cyprus

**Keywords:** severe acute respiratory syndrome coronavirus 2, COVID-19, variant of concerns, reverse transcription quantitative polymerase chain reaction, molecular diagnosis

## Abstract

Variants (Alfa, Gamma, Beta, and Delta) of severe acute respiratory syndrome coronavirus 2 (SARS-CoV-2) are circulating worldwide. These variants of concerns share some common mutations but they also have distinguishing mutations. These mutations affect transmissibility of virus and cause evasion from neutralizing antibodies. Monitoring and identification of circulating variants is of great importance for public health. In this study, an in-house SARS-CoV-2 reverse transcription quantitative polymerase chain reaction (RT-qPCR) kit was designed to detect variants of concerns by the World Health Organization.

Primer sets and probes were designed to target presence of virus along with mutations for identifying different variants (for N501Y, HV69–70del, K417N, and T478K). Reactions were set by using commercially available master mixes without a reference dye.

The RT-qPCR conditions were optimized by using commercially available ribonucleic acid samples of wild-type, Alfa, Beta, Gamma, and Delta variants. Several samples were also analyzed by the in-house kit after optimization studies. All Alfa variant and wild-type samples were also double confirmed with a commercially available variant detection kit demonstrating a 100% consistence with the in-house kit. Beta, Gamma, and Delta variants could not be confirmed with any other commercially available kits as there is not any available one in the market.

SARS-CoV-2 variants are gaining importance during the pandemic and shaping the fight against the virus. RT-qPCR kits detecting different variants would provide a significant advantage while screening the population.

## Introduction

Emergence of novel variants of severe acute respiratory syndrome coronavirus 2 (SARS-CoV-2) is raising concerns worldwide. Especially the increase in prevalence of the spike N501Y mutation containing SARS-CoV-2 variants, namely Alfa (B.1.1.7 or VOC-202012/01, United Kingdom), Gamma (P.1, Brazil), and Beta (B.1.351, South Africa) gained significant importance over the last 5 months since the authorities of the United Kingdom reported that SARS-CoV-2 variant of concern (VOC) 202012/01 or B.1.1.7 took over other lineages across the country within a few weeks. 1 2 These lineages contain several mutations especially clustered in the spike gene. N501Y substitution in the spike protein is located at the receptor-binding motif and was shown to increase the binding affinity of virus to the host angiotensin-converting enzyme 2 receptor. 3 4 Therefore, this particular mutation is responsible for higher transmissibility reported for the Alfa lineage, as well as the Beta and Gamma lineages. 5 6

Even though all of these three variants that arose significant concern worldwide share the N501Y mutation, they also harbor other distinguishing mutations. The HV69–70del mutation in the N-terminal domain of spike protein is one of the several mutations present in the Alfa strain. Copresence of this mutation with N501Y substitution identifies the Alfa variant. This deletion was associated with immune escape in patients with immunocompromised situation. 1 7 Beta and Gamma variants harbor the N501Y mutation but do not contain the HV69–70del. Instead, these two variants contain the E484K substitution, which may allow evasion from neutralizing antibodies. 8 The variation that can distinguish the Beta and Gamma variants is the K417N substitution, which is present in the Gamma lineage. Molecular dynamic simulations have shown that the combination of N501Y with E484K and K417N may induce greater conformational changes in the spike protein when compared with N501Y alone. 9

In late 2020 a new VOC, which does not harbor the N501Y mutation but is more contagious than previous variants, was detected in India and was named as the Delta (B.1.617.2) variant. 10 Delta variant harbors T478K, P681R, and L452R spike protein substitutions that can affect transmissibility and also may confer evasion from current vaccine antibodies.

Emergence of new SARS-CoV-2 variants is continuing to raise concerns as they significantly affect transmissibility, disease severity, and antibody evasion. In this context, continuous, robust, and widespread SARS-CoV-2 surveillance and monitoring of circulating variants within the population is crucial. The aim of this study is to develop a SARS-CoV-2 reverse transcription quantitative polymerase chain reaction (RT-qPCR) kit to detect VOCs by the World Health Organization.

## Material and Methods

### Primer and Probe Design

Gene-specific primer sets and probes were designed to target nucleocapsid 1 (N1), nucleocapsid 2 (N2), and open reading frame (ORF) genes to detect the presence of virus in oro-/nasopharyngeal swab specimens. Mutation-specific primers and probe sequences were designed for N501Y, HV69–70del, K417N, and T478K mutation regions. HV69–70del primers were designed for the wild-type genotype, while others targeted the mutant genotypes. NC_045512.2 was used as the SARS-CoV-2 reference genome sequence for primer probe design. 11 Gene-specific primers and probe were designed to target the RP gene on human genome as an internal control (IC) to detect the presence of human epithelial cells in oro-/nasopharyngeal swab specimens. NC_000010.11, GRCh38.p13 primary assembly was used as the reference sequence for human genome. Informed consent has been taken from the individuals who voluntarily participated for the optimization of the *in-house* kit.

### Ribonucleic Acid Extraction Protocol

SARS-CoV-2 wild-type, Alfa, Beta, Gamma, and Delta variants' ribonucleic acid (RNA) extraction was performed by using a commercially available nucleic acid extraction kit (Viral DNA and RNA Extraction Kit;GenRotex96, TianLong, China). For this purpose, 200 mL of the viral transport medium solution containing oro-/nasopharyngeal swab was transported into the wells of the kit for isolation. In the end of the protocol, yield of viral RNA was 50 μL ( Table 1 ).

**Table 1:** Specific primers and probes sequences designed to detect HV69–70 deletion, N501Y, K417N, and T478K mutations

Gene	Amino acid change	Primers	Nucleotide sequence	Tm (°C)	Product size (base pair, bp)
SARS-CoV-2 Spike (S)	HV 69–70 deletion	Forward primer	GTTACTTGGTTCCATGCTATACATG	57	90
		Reverse primer	CCAATGTTACTTGGTTCCATGCTATAC	59	
		Probe	Texas Red-CTCTGGGACCAATGGTACTAAGAGGTTTG-BHQ2	62	
	N501Y	Forward primer	ATCATATGGTTTCCAACCCACTT	55	90
		Reverse primer	GCTGGTGCATGTAGAAGTTC	57	
		Probe	Texas Red-GGTTACCAACCATACAGAGTAGTAGTACTTTC-BHQ2	60	
	K417N	Forward primer	CTCCAGGGCAAACTGGAAAG	57	110
		Reverse primer	CCACCAACCTTAGAATCAAGATTGTTAG	60	
		Probe	Texas Red-CCAGATGATTTTACAGGCTGCGTTATAGCT-BHQ2	62	
	T478K	Forward primer	CTATCAGGCCGGTAGCAA	58	116
		Reverse primer	CTACTCTGTATGGTTGGTAACC	55	
		Probe	Texas Red-CCTTTACAATCATATGGTTTCCAACCCACTWATGG-BHQ2	62	

Abbreviations: SARS-CoV-2, severe acute respiratory syndrome coronavirus 2; Tm, temperature.

### Reaction Setup Optimization of RT-qPCR Conditions

Commercially available RT-qPCR ready master mixes (Hibrigen, Gebze, Kocaeli, Turkey), without a reference dye were used according to the manufacturer's instructions. All primer and probe sets were optimized in Insta Q96 Plus Real-time PCR Detection System (HiMedia Laboratories Pvt. Ltd., India) and Rotor Gene-Q (Qiagen N.V., Germany) using commercially available wild-type, Alfa, Beta, Gamma, and Delta variant RNA samples from AmpliRun (Vircell, Spain). Details of the reaction setup and the two-step RT-qPCR program are given in Table 2 .

**Table 2:** Cycling conditions for the primer and probe sets are represented in the table

RT-qPCR program		
Cycle (s)	Temperature	Duration
1	55°C	05:00
1	95°C	00:30
40	95°C	00:05
	60°C	00:10

Abbreviation: RT-qPCR, reverse transcription quantitative polymerase chain reaction.

Note: Read on FAM, HEX, and Texas Red channels.

Cycle threshold (Ct) level is set automatically by the software. The samples that were assigned with a Ct value by the instrument should have sigmoidal curves. If the samples do not have sigmoidal curves their results are considered negative. If the curves are sigmoidal and the sample has an assigned Ct value, the value is used for further analysis.

ICs are included to detect the presence of human epithelial cells in the oro-/nasopharyngeal swab specimens. All reactions with RNA samples should give a positive Ct value for IC. If a sample does not have a Ct value for the IC, the reaction should be repeated.

Negative or no template controls and positive controls must be included in every reaction. No positive Ct values are expected in negative or no template controls. Ct values in these samples indicate contamination, and therefore all reactions should be repeated. Positive controls should have positive Ct values. No Ct values in positive controls suggest that the reaction did not work, so all reaction should be repeated in this case as well.

Expected results for the wild-type and the variants with the designed kit are shown in Table 3 .

**Table 3:** Expected results from the RT-qPCR analysis for each variant, with designed primer probe sets are represented in the table

		Variant type			
	Wild-type	United Kingdom (Alfa)	South Africa (Beta)	Brazil (Gamma)	India (Delta)
HV 69–70 deletion	+	–	+	+	+
N501Y	–	+	+	+	–
K417N	–	+	–	+	+
T478K	–	–	–	–	+
N1 + N2 + Orf1ab	+	+	+	+	+
RP	+	+	+	+	+

Abbreviation: RT-qPCR, reverse transcription quantitative polymerase chain reaction.

Note: Variant analysis is performed according to this table.

## Results

RT-qPCR conditions were optimized using commercially available wild-type, Alfa, Beta, Gamma, and Delta variant RNA samples. Upon optimization, selected SARS-CoV-2 positive samples were also analyzed using the designed kit with the given conditions.

**Fig. 1:**
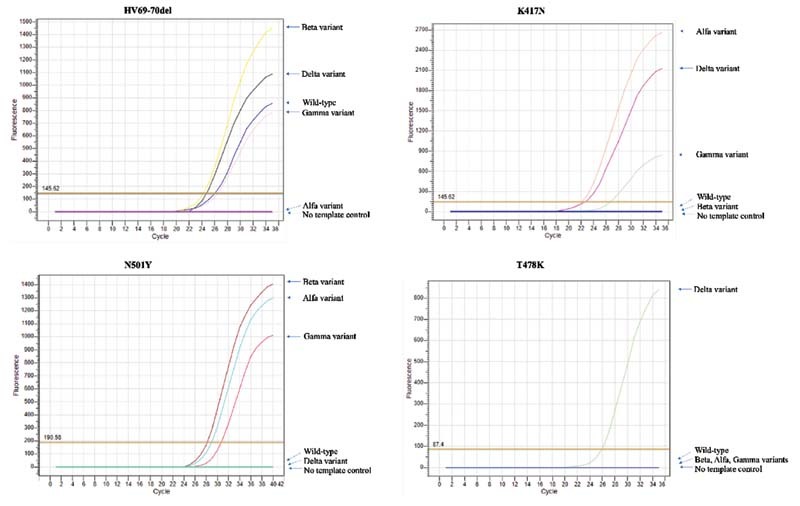
Fluorescence curves for HV69–70del primer and probe set (upper left panel), N501Y primer and probe set (lower left panel), K417N primer and probe set (upper right panel), and T478K primer and probe set (lower right panel) are represented in the figure. Samples that are below the threshold level (shown as the horizontal brown line) are considered negative, whereas samples that have fluorescence above the threshold level are positive for the specific primer and probe set.

Fig. 1 represents the fluorescence curves obtained in each primer probe set for each variant, wild-type SARS-CoV-2, and a no template control reaction.

UK variant and wild-type samples were also studied in parallel with another commercial kit, SARS-CoV-2 Variant Real Time PCR Kit by SARS-CoV-2 Variant Real Time PCR Kit (Cat No: 114R-10–04, SNP Biotechnology R&D Ltd., Ankara, Turkey) as well as UK and Delta variants samples were confirmed by Hibrigen Biotechnology R&D Industry and Trade Inc. (Gebze, Kocaeli, Turkey) RT-PCR kits according to the manufacturer's guidelines. Results were 100% compatible in both kits.

**Fig. 2:**
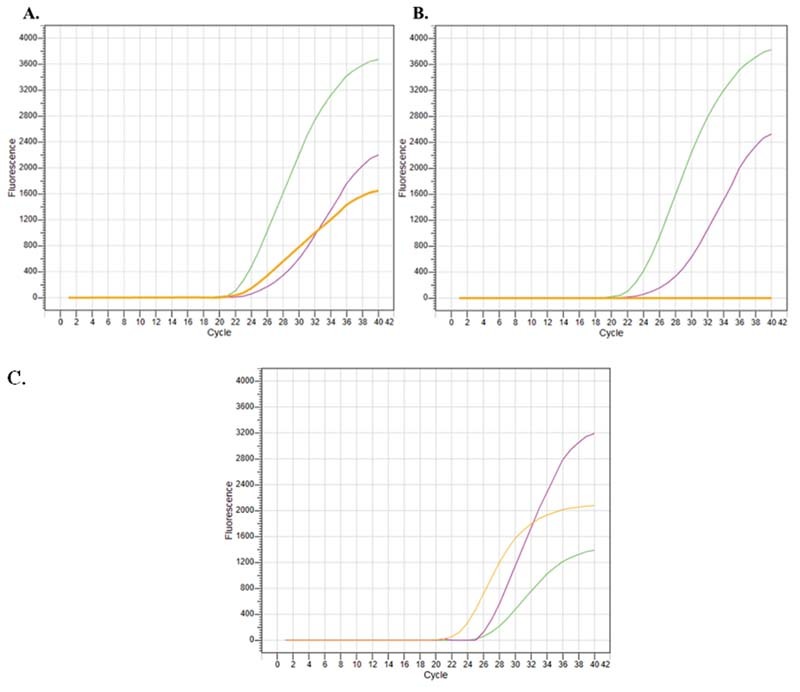
Results of a UK variant sample by the designed kit. ( **A** ) N501Y detection. ( **B** ) 69–70del detection. Pink: HEX channel/internal control; Green: FAM channel/viral genome; Orange: CY5 channel/mutation. ( **C** ) Results of a UK variant sample with the commercial kit (SARS-CoV-2 Variant Real Time PCR Kit). Pink: HEX channel/internal control; Green: FAM channel/N501Y mutation; Orange: CY5 channel/69–70del mutation.

Fig. 2 represents the results obtained from an UK variant sample with the *in-house* designed kit. In both panels the green curve indicates the presence of SARS-CoV-2 viral genome (N1 + N2 + Orf1ab), which means that the individual is SARS-CoV-2 positive, and the pink curve indicates the IC (RP), which confirms adequate sampling. Presence of N501Y substitution is detected by the N501Y primers and is indicated by the orange curve in Fig. 2A . In Fig. 2B , results of the HV69–70del detection in the same sample is indicated by the orange line. As the primers for the 69–70 deletion was designed to target the wild-type genome, no amplification is observed in the UK variant samples. Same sample was further analyzed with the commercial kit and consistent results were obtained as represented in Fig. 2C .

**Fig. 3:**
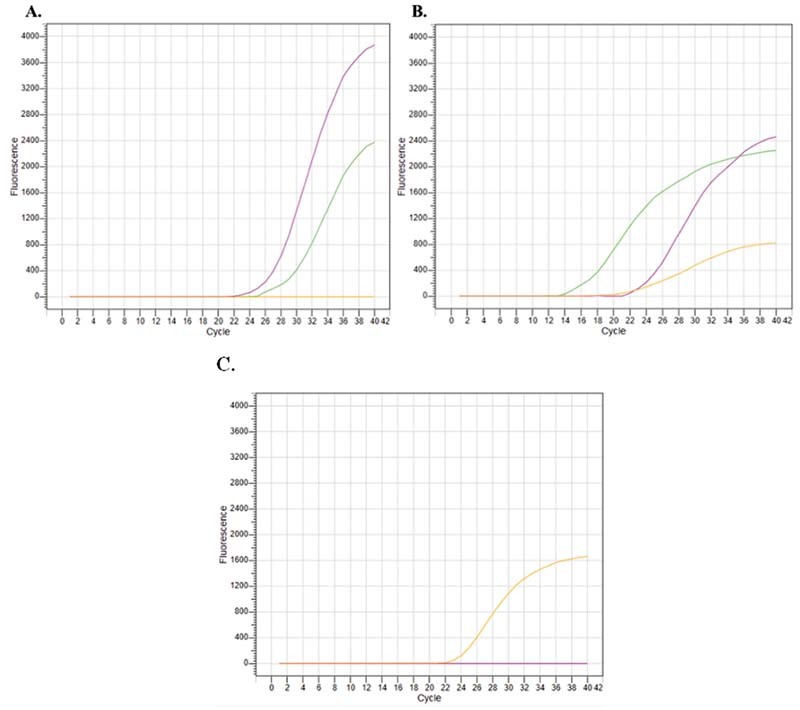
Results of a wild-type severe acute respiratory syndrome coronavirus 2 (SARS-CoV-2) positive sample by the designed kit ( **A** and **B** ). ( **A** ) N2 + N1 + Orf1ab (FAM channel/green), RP (HEX channel/pink), N501Y mutation (Texas Red channel). ( **B** ) N2 + N1 + Orf1ab (FAM channel/Green), RP (HEX channel/pink), HV69–70del primers for wild-type (Texas Red channel). ( **C** ) Results of a wild-type SARS-CoV-2 positive sample by commercial kit (SNP Biotechnologies), orange: CY5 channel/internal control.

Fig. 3 represents the results obtained with a wild-type sample. Green and pink lines are the SARS-CoV-2 (N2 + N1 + Orf1ab) and IC (RP) amplification curves, respectively. As wild-type SARS-CoV-2 does not harbor the N501Y substitution, no amplification occurred ( Fig. 3A , orange line). HV69–70del primers, on the other hand, recognize the wild-type sequence and amplification occurs ( Fig. 3B , orange line). Same sample was then further analyzed with the commercial kit and consistent results were obtained as represented in Fig. 3C .

## Discussion

Emergence of novel variants of SARS-CoV-2 is a huge concern across the World. B1.1.7 known as UK variant has gained a lot of importance as it contains a spike mutation (N501Y) which is increasing the transmissibility of the virus. 5 6 N501Y is not the only spike mutation of UK variant, HV69–70del mutation on the N-terminal domain is another mutation. N501Y mutation is common in some other variants including P.1 and B.1.351; however, HV69–70del mutation is the mutation allowing us to distinguish between these variants. 1 7 So, for this purpose, RT-qPCR kits detecting these mutations specific for each variant gained a lot of importance while having decisions during pandemic.

The kit designed was specific for detecting several variants of concern including Alfa, Beta, Gamma, and Delta variants which are current variants of concerns. There are number of PCR kits in the market detecting two mutations (N501Y and HV69–70del) for Alfa variant, and one of them is SNP. A comparison study between the kit designed and a commercial kit was performed to investigate the reliability of the designed kit with a commercial one. The data indicated that, all the samples studied in both kits were 100% consistent with each other, showing the reliability of the designed kit, over the kits in market.

There are a few differences between the in-house kit and the commercial kits. The SARS-CoV-2 Variant Real Time PCR Kit (SNP Biotechnology R&D Ltd.) provides detection of both mutations in single run; however, the absence of the primers for detecting the SARS-CoV-2 viral genome is one of the differences. The in-house kit is detecting both N501Y and HV69–70del mutations, as well as the viral genome. So, this provides the kit a great advantage for detecting the mutant variants while screening the population.

The samples of Beta, Gamma, and Delta were successfully detected by using the in-house kit; however, a comparison study could not be performed with a commercial kit as there are no other commercially available kits detecting Beta, Gamma, and Delta variants.

Overall, the prevalence of SARS-CoV-2 variants is gaining huge importance during the pandemic and shaping the fight against this virus, these kits detecting the viral genome as well as the mutations would be really significant while having decisions.
